# CTHRC1 expression is a novel shared diagnostic and prognostic biomarker of survival in six different human cancer subtypes

**DOI:** 10.1038/s41598-021-99321-w

**Published:** 2021-10-06

**Authors:** Nuzhat Sial, Mukhtiar Ahmad, Muhammad Safdar Hussain, Muhammad Junaid Iqbal, Yasir Hameed, Mehran Khan, Mustansar Abbas, Rizwan Asif, Jalil Ur Rehman, Muhammad Atif, Muhammad Rashid Khan, Zahid Hameed, Hina Saeed, Rida Tanveer, Saba Saeed, Aneeqa Sharif, Hafiz Muhammad Asif

**Affiliations:** 1 Department of Zoology, The Islamia University of Bahawalpur, Bahawalpur, Pakistan; 2 Department of Biochemistry and Biotechnology, The Islamia University of Bahawalpur, Bahawalpur, 63100 Pakistan; 3 Department of Biosciences, COMASTS University Islamabad, Islamabad, Pakistan; 4 Department of Pharmacy, The Islamia University of Bahawalpur, Bahawalpur, Pakistan; 5 Department of Eastern Medicine, Government College University Faisalabad, Faisalabad, Pakistan; 6 Department of Microbiology, Government College University Faisalabad, Faisalabad, Pakistan; 7 University College of Conventional Medicine, Faculty of Pharmacy and Alternative Medicine, The Islamia University of Bahawalpur, Bahawalpur, Pakistan; 8 University College of Eastern Medicine, The Islamia University of Bahawalpur, Bahawalpur, Pakistan; 9 Department of Biological Sciences, International Islamic University, Islamabad, Pakistan; 10 Department of Zoology, University of the Punjab, Lahore, Pakistan; 11 Department of Zoology, Mirpur University of Science and Technology, Mirpur, Pakistan

**Keywords:** Biotechnology, Cancer, Cell biology, Computational biology and bioinformatics

## Abstract

According to the previous reports, the collagen triple helix repeat containing 1 (CTHRC1) causes tumorigenesis by modulating the tumor microenvironment, however, the evidence is limited to a few human cancer subtypes. In the current study, we analyzed and validated the CTHRC1 expression variations in 24 different human cancer tissues paired with normal tissues using publically available databases. We observed that CTHRC1 was overexpressed in all the 24 major subtypes of human cancers and its overexpression was significantly associated with the reduced overall survival (OS) duration of head and neck squamous cell carcinoma (HNSC), kidney renal clear cell carcinoma (KIRC), liver hepatocellular carcinoma (LIHC), Lung adenocarcinoma (LUAD), stomach adenocarcinoma (STAD), and Uterine corpus endometrial carcinoma (UCEC). This implies that CTHRC1 plays a significant role in the development and progression of these cancers. We further noticed that CTHRC1 was also overexpressed in HNSC, KIRC, LIHC, LUAD, STAD, and UCEC patients of different clinicopathological features. Pathways enrichment analysis revealed the involvement of CTHRC1 associated genes in seven diverse pathways. We also explored few interesting correlations between CTHRC1 expression and promoter methylation, genetic alterations, CNVs, CD8+ T immune cells infiltration, and tumor purity. In conclusion, CTHRC1 can serve as a shared diagnostic and prognostic biomarker in HNSC, KIRC, LIHC, LUAD, STAD, and UCEC patients of different clinicopathological features.

## Introduction

Cancer is one of the primary killers Worldwide^1^. In 2020, around 1.8 million new cancer cases and 0.6 million cancer-related deaths were recorded in the United States (US) alone^2^. Late diagnosis, poor prognosis, and metastasis are the main causes of the high mortality rate due to cancer^3^. Despite tremendous advances in chemotherapy and surgical resection, the prognosis of cancer patients still remains low, with an average 5-year survival rate of around 5–10% in most of the cancer subtypes^4^. Therefore, the discovery of novel shared diagnostic and prognostic molecular markers will be helpful in managing the disease and improving the treatment outcomes.

Collagen triple helix repeat containing 1 (CTHRC1), a 30 kDa protein is generally expressed in developing bones, cartilage, and myofibroblasts during wound healing^5^. Earlier studies have reported the dysregulation of CTHRC1 in different human cancers including cervical carcinoma^6^, non-small cell lung cancer^7^, endometrial cancer^8^, colorectal, breast, and prostate cancer^9–11^. Moreover, Ke et al. have also confirmed that overexpression of CTHRC1 at translational level is significantly associated with the reduced overall survival (OS) duration of the non-small cell lung cancer (NSCLC) patients, but the potential causes behind CTHRC1 up-regulation was not elucidated in this study^7^. However, to the best of our knowledge, CTHRC1 dysregulation and its impact on the various other subtypes of cancer is yet to uncover. Therefore, the main aim of our study is to comprehensively investigate CTHRC1 in distinct other subtypes of human cancers and find more closely CTHRC1-associated cancer subtypes through an integrated analyses approach.

For this purpose, we used UALCAN, GENT2, and HPA databases, as well as the Kaplan–Meier (KM) plotter tool to analyze and validate CTHRC1 expression and its correlation with the prognosis of distinct cancer subtypes. Furthermore, we utilized the TIMER database to find the Spearman correlation between CTHRC1 expression and CD8+ T cells infiltration and tumor purity. Additionally, we also explored the CTHRC1-associated genetic alterations, copy number variations (CNVs), DNA methylation, pathways, and gene–drug network. Taken together, our results indicated that CTHRC1 can be a potential diagnostic and prognostic biomarker and therapeutic target in head and neck squamous cell carcinoma (HNSC), kidney renal clear cell carcinoma (KIRC), liver hepatocellular carcinoma (LIHC), Lung adenocarcinoma (LUAD), stomach adenocarcinoma (STAD), and Uterine corpus endometrial carcinoma (UCEC).

## Methods

### UALCAN

UALCAN (http://ualcan.path.uab.edu) webserver is based on the TCGA OMICS data and is frequently used for expression profiling of the gene(s) of interest across more than 20 cancer subtypes. In our study, transcription expression and methylation profiling of CTHRC1 expression profiles across multiple cancer subtypes was evaluated via UALCAN^12^. All the analyses were run with the default settings in UALCAN. For statistics, this tool employs student t-test and normalized mRNA expression level as transcript per million (TPM) reads while the level of promoter methylation as beta (β) value. A p-value of < 0.05 was regarded statistically significant.

### Kaplan–Meier plotter

Kaplan–Meier plotter (http://kmplot.com/analysis/)^13^ is an intuitive online tool for analyzing prognostic values of the gene(s) of interest in more than 20 cancer subtypes. This resource includes the survival data of approximately 54,675 genes derived from 10,461 cancerous samples of 30 different cancer subtypes. We carried out the overall survival (OS) analysis of CTHRC1 in distinct cancer subtypes using this tool. To access the OS, based on gene expression, cancer patients were divided into two different groups (high and low expression). Hazard ratios (HRs), 95% confidence intervals (CIs), and the log-rank p-value were also computed and displayed.

### The OShnscc, OSkirc, OSlihc, OSluca, OSucec, and GEPIA databases

Different freely available online consensus survival analysis databases including OShnscc^14^, OSkirc^15^, OSlihc^16^, OSluca^17^, OSucec (http://bioinfo.henu.edu.cn/DatabaseList.jsp)^14^, and GEPIA (http://gepia.cancer-pku.cn/)^18^ were used to further validate the prognostic value of CTHRC1 in HNSC, KIRC, LIHC, LUAD, STAD, and UCEC. These databases acquired expression and survival information from the Gene Expression Omnibus (GEO) based datasets. During the analysis, we simply queue the CTHRC1 gene in these databases to acquire the KM OS plots with hazard ratios (95%).

### GENT2 database

GENT2 (http://gent2.appex.kr/) is a cancer transcriptomics data analysis webserver^19^. The NCBI GEO database (https://www.ncbi.nlm.nih.gov/geo/) is a source of transcriptomics data for GENT2, that is acquired by the Affymetrix U133A and U133 Plus2 microarray platforms. In the current study, we used this database for the transcription expression validation of CTHRC1 using independent cohorts of distinct cancer patients. For statistics, this tool use student t-test. A p-value of < 0.05 was regarded statistically significant.

### Immunohistochemistry (IHC) staining

To analyze the differences in CTHRC1 proteomics expression level, CTHRC1 IHC images of proteomics expression in normal controls and six cancerous tissues (Head and neck cancer, kidney cancer, liver cancer, lung cancer, stomach cancer, and endometrial cancer) were taken from the HPA (Human Protein Atlas) database (http://www.proteinatlas.org/)^20^ and further analyzed. The observed proteomics expression level was graded as not detected, low, medium, and high, based on the intensity of staining and fraction of the stained cells.

### cBioportal

cBioPortal (http://www.cbiobortal.org) database was created by Memorial Sloan Kettering Cancer Center (MSK) for analyzing TCGA genomic data of more than 30 cancer subtypes^21^. In our study, genetic mutational and copy number variations (CNVs) profiles of CTHRC1 in distinct human cancer subtypes were obtained using this database.

### PPI network construction, visualization, and pathway enrichment analysis

In our study, we utilized the STRING database (https://string-db.org/)^22^ for subsequently analyzing the protein–protein interaction (PPI) network of CTHRC1. The main parameters for this PPI construction included: minimum required interaction score [“Low confidence (0.150)”], max numbers of interactors to show (“no more than 50 interactors” in 1st shell), and active interaction sources (“experiments”). The obtained PPI network was then visualized using Cytoscape software 3.7.2^23^ and the pathway enrichment analysis of the CTHRC1 network genes was performed through an online tool, DAVID (available at: http://david.ncifcrf.gov/summary.jsp)^24^. A p-value < 0.05 was considered as significant.

### Tumor Immune Estimation Resource (TIMER) Database

Tumor Immune Estimation Resource (TIMER) (http://timer.cistrome.org/) database in-house approximately 10,000 samples of more than 30 cancer subtypes from TCGA projects. It is an easy-to-use tool for systematically analyzing the Spearman correlation between immune infiltrate, tumor purity and expression level of the gene(s) of interest^25^. In this study, we evaluated the Spearman correlation between CTHRC1 expression and CD8+ T immune cells infiltration and tumor purity in distinct cancer subtypes using the TIMER database. A p-value < 0.05 was considered as significant.

### CTHRC1 gene–drug interaction network analysis

In CTHRC1 drug–gene interaction network, we identified those drugs that interact with CTHRC1 and increase or decrease its mRNA expression using CTD (http://ctdbase.org/) database and Cytoscape software^23^. The CTD database is a resource of physically curated drug–gene interactions from the literature^26^.

## Results

### CTHRC1 transcriptional expression in human cancers

**Figure 1:**
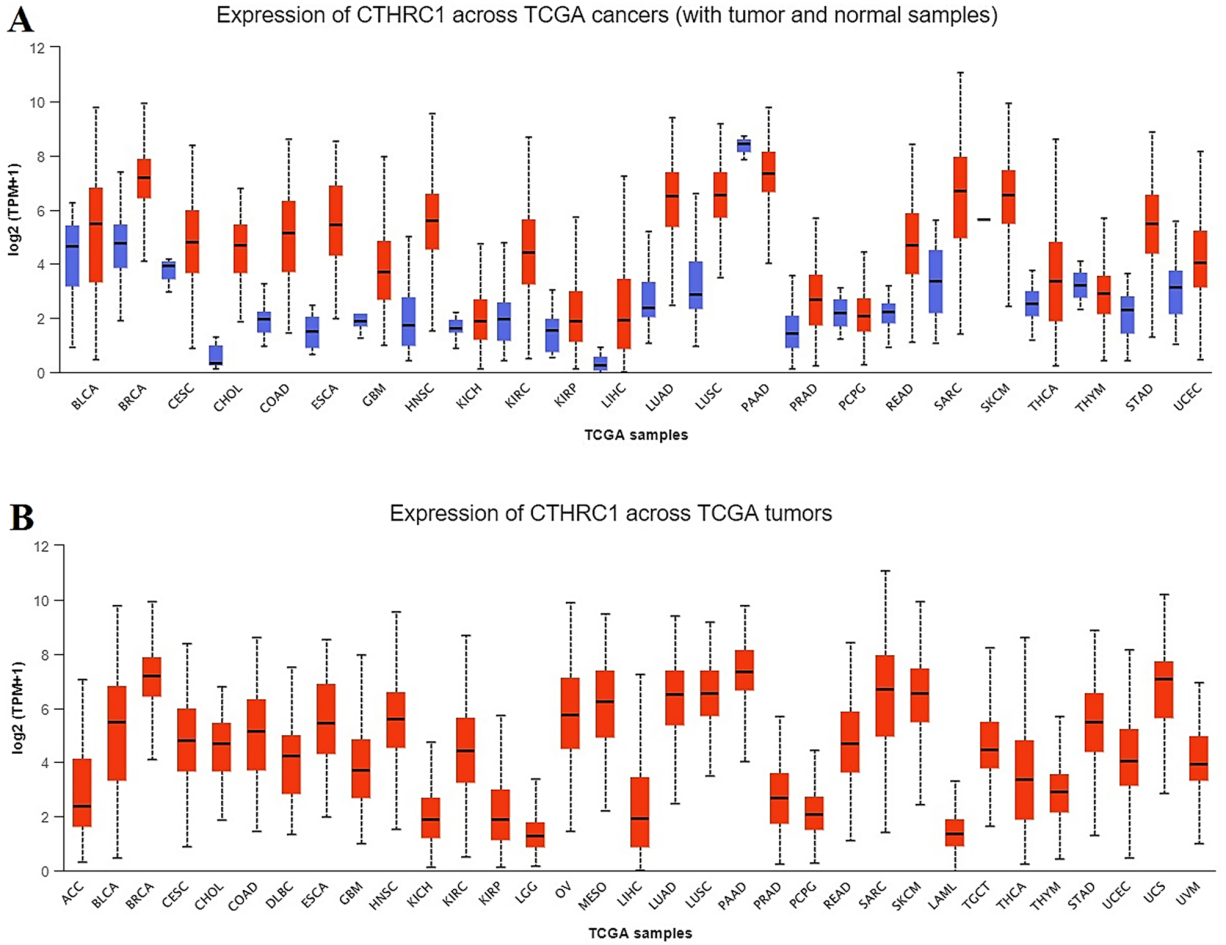
CTHRC1 transcription expression level in 24 human cancer subtypes. (**A**) CTHRC1 expression across cancerous samples paired with normal controls, and (**B**) CTHRC1 expression across cancerous samples without paired normal controls (p < 0.05).

By exploring the UALCAN databases, we analyzed the CTHRC1 expression across 24 different cancer tissues paired with normal samples. Our results revealed the notably elevated CTHRC1 transcriptional level in all these cancer tissues relative to normal control including head and neck squamous cell carcinoma (HNSC), kidney renal clear cell carcinoma (KIRC), liver hepatocellular carcinoma (LIHC), Lung adenocarcinoma (LUAD), stomach adenocarcinoma (STAD), and Uterine corpus endometrial carcinoma (UCEC) (Fig. 1).

### CTHRC1 prognostic values in six different types of human cancers

**Figure 2:**
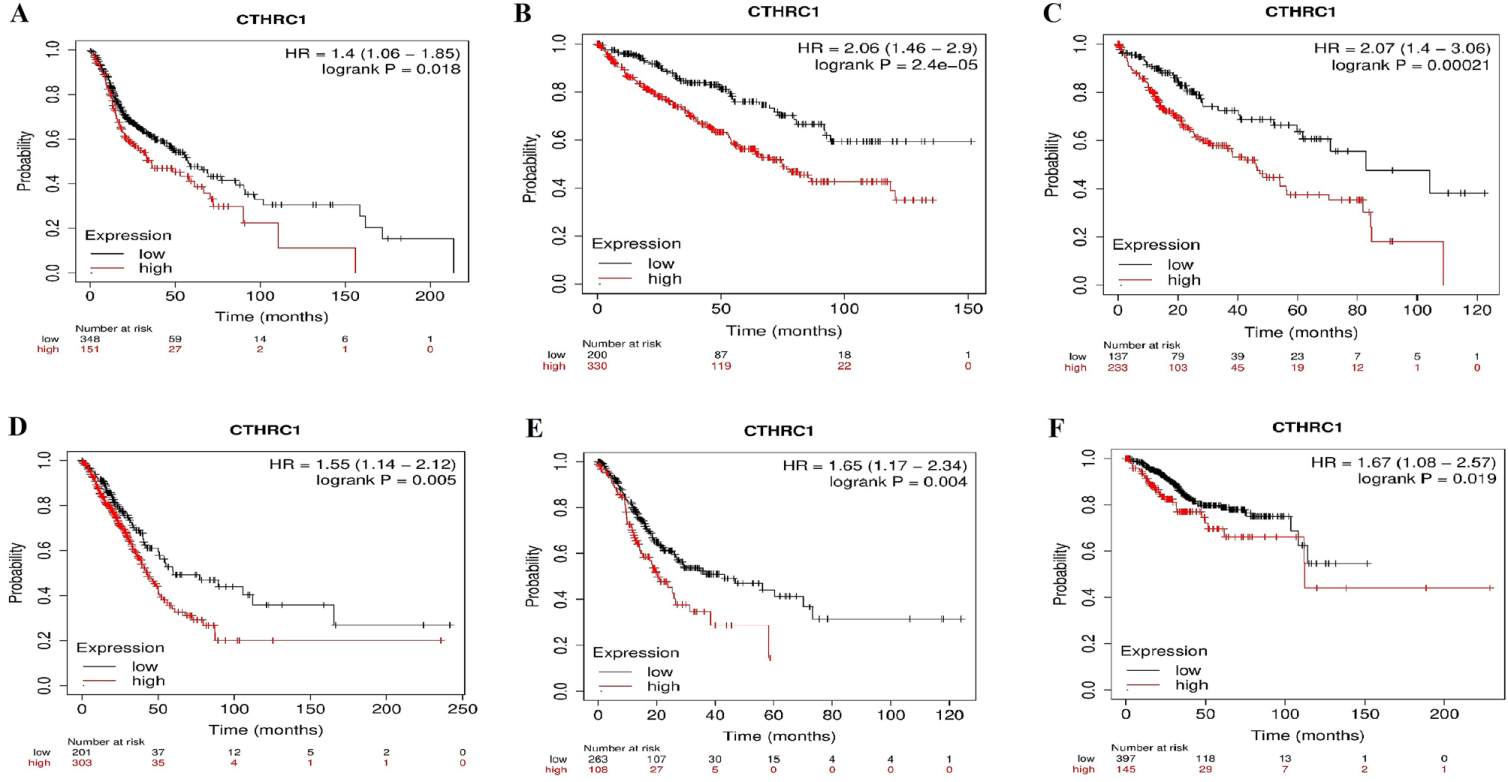
Relationship between CTHRC1 expression and OS duration of the distinct types of cancers. (**A**) HNSC, (**B**) KIRC, (**C**) LIHC, (**D**) LUAD, (**E**) STAD, and, (**F**) UCEC. A p-value of < 0.05 was selected as a cutoff criterion.

Via KM plotter tool, next, we investigated whether CTHRC1 higher transcriptional level was associated with the OS duration of the cancer patients or not. We observed that higher CTHRC1 transcriptional level significantly (p > 0.05) reduced the OS duration of the patients HNSC (HR = 1.4, 95% CI 1.05–1.85, p = 0.018), KIRC (HR = 2.06, 95% CI 1.46–2.9, p = 2.4e − 05), LIHC (HR = 2.07, 95% CI 1.4–3.06, p = 0.00021), LUAD (HR = 1.55, 95% CI 1.14–2.12, p = 0.005), STAD (HR = 1.65, 95% CI 1.17–2.34, p = 0.004), and UCEC (HR = 1.67, 95% CI 1.08–2.57, p = 0.00021) patients (Fig. 2).

### Validation of the CTHRC1 prognostic values in HNSC, KIRC, LIHC, LUAD, STAD, and UCEC using independent cohorts via OShnscc, OSkirc, OSlihc, OSluca, OSucec, and GEPIA databases

**Figure 3:**
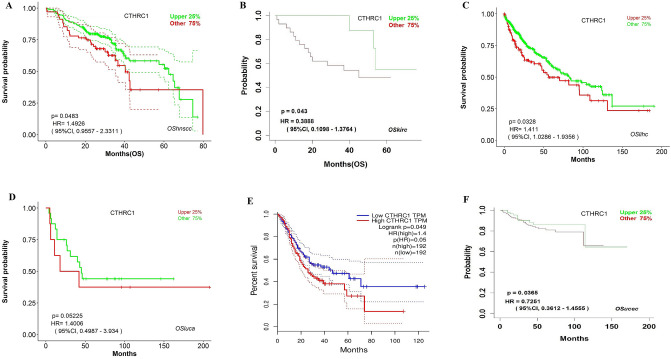
The validation of CTHRC1 prognostic values in HNSC, KIRC, LIHC, LUAD, STAD, and UCEC. (**A**) Prognostic value of CTHRC1 in HNSC using OShnscc database, (**B**) Prognostic value of CTHRC1 in KIRC using OSkirc database, (**C**) Prognostic value of CTHRC1 in LIHC using OSlihc database, (**D**) Prognostic value of CTHRC1 in LUAD using OSluca database, (**E**) Prognostic value of CTHRC1 in STAD using GEPIA database, and, (**F**) Prognostic value of CTHRC1 in UCEC using OSucec database. The red color in Kaplan–Meier plots shows the higher expression of CTHRC1 while green and blue color indicates the lower expression. The x-axis represents survival time and the y-axis represents survival rate.

For validating the prognostic values of CTHRC1 in KIRC in HNSC, KIRC, LIHC, LUAD, STAD, and UCEC using new independent cohorts, we utilized a variety of publically accessible online tools including OShnscc (for validating the CTHRC1 prognostic value in HNSC), OSkirc (for validating the CTHRC1 prognostic value in KIRC), OSlihc (for validating the CTHRC1 prognostic value in LIHC), OSluca (for validating the CTHRC1 prognostic value in LUAD), OSucec (for validating the CTHRC1 prognostic value in UCEC), and GEPIA (for validating the CTHRC1 prognostic value in STAD). The analysis results by these tools have validated the findings of KM plotter and demonstrated that higher expression of CTHRC1 is associated with the reduced OS duration of the, HNSC (HR = 1.4926, 95% CI 0.9557–2.3311, p = 0.0483), KIRC (HR = 0.3888, 95% CI 0.1098–1.3764, p = 0.043), LIHC (HR = 1.411, 95% CI 1.0286–1.9356, p = 0.0328), LUAD (HR = 1.4006, 95% CI 0.4987–3.934, p = 0.5225), STAD (HR = 1.4, 95% CI p = 0.049), and UCEC (HR = 0.7251, 95% CI 0.3612–1.4555, p = 0.0365) (Fig. 3). Taken together the results of CTHRC1 prognostic value analysis, it was observed that the higher expression level of SCTHRC1 is vital in the tumorgenesis of the HNSC, KIRC, LIHC, LUAD, STAD, and UCEC. Therefore the next part of our study will mainly focus on the unique role of CTHRC1 in these six types of human cancers.

### Correlation between CTHRC1 transcriptional expression level and different clinicopathological features of HNSC, KIRC, LIHC, LUAD, STAD, and UCEC

**Figure 4:**
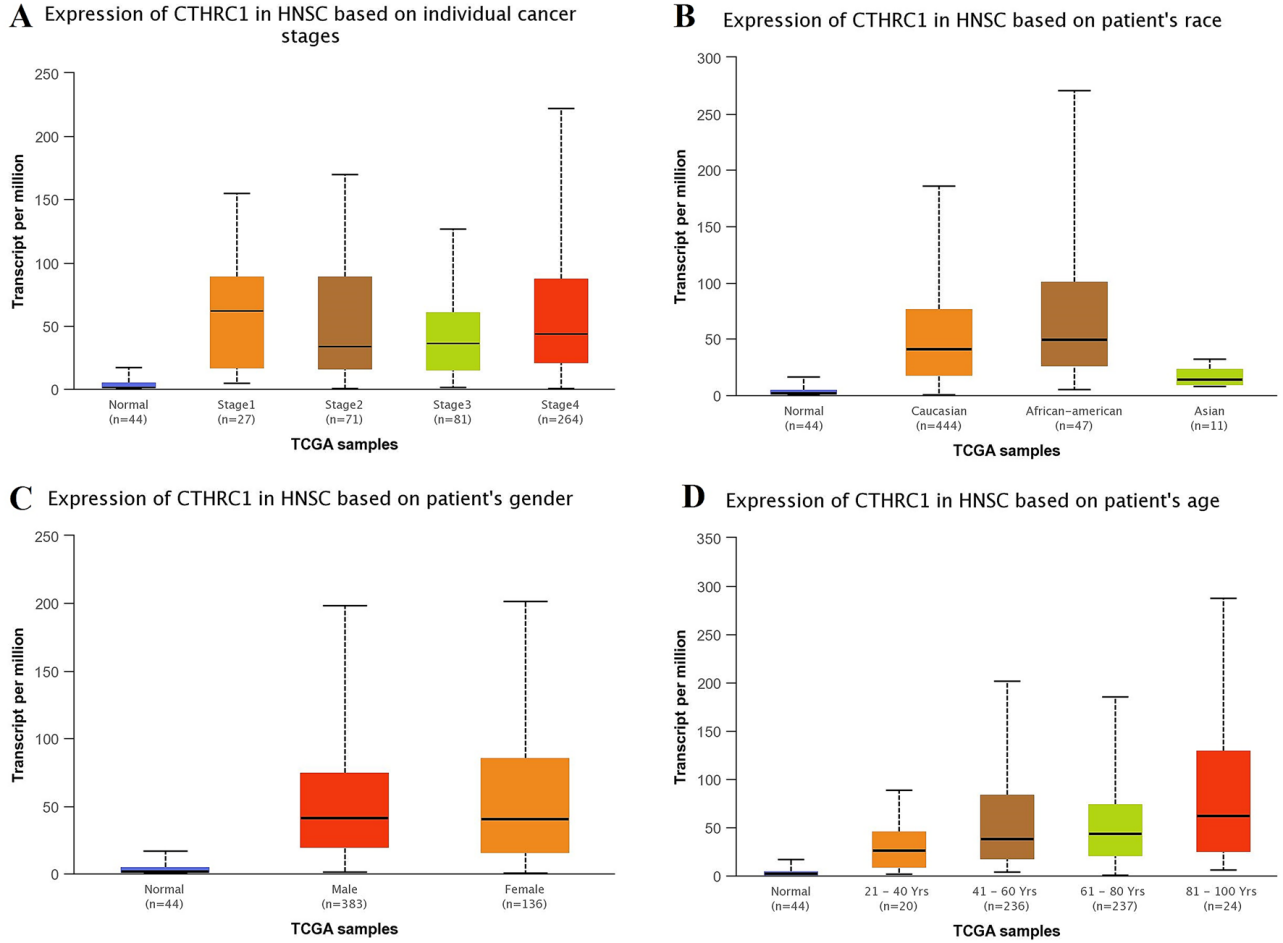
CTHRC1 transcription expression in various clinicopathological parameters of HNSC. (**A**) Different cancer stages based, (**B**) Different patients race based, (**C**) Different patients gender based, and (**D**) Different age groups based. A p-value of < 0.05 was selected as a cutoff criterion.

**Figure 5:**
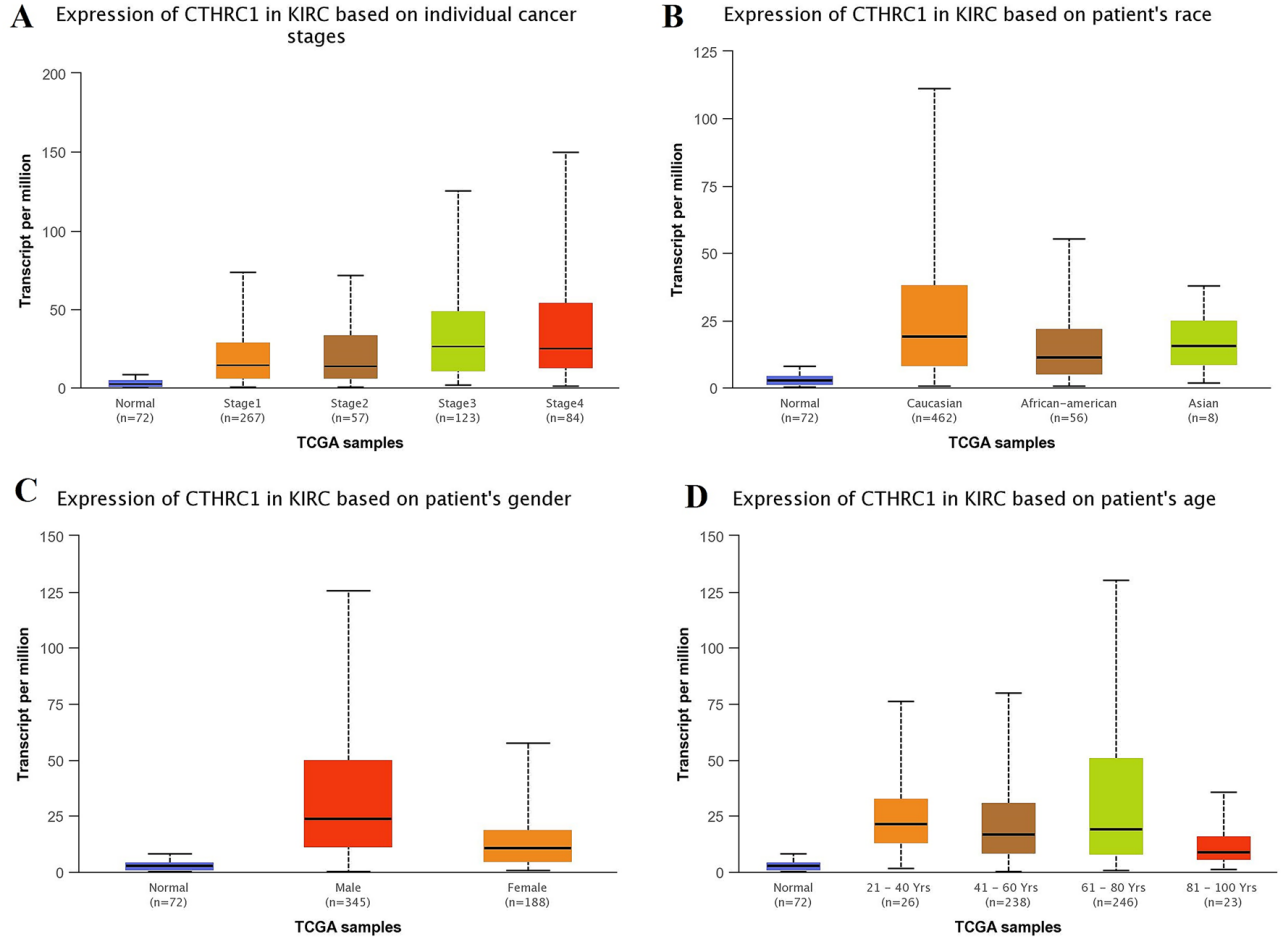
CTHRC1 transcription expression in various clinicopathological parameters of KIRC. (**A**) Different cancer stages based, (**B**) Different patients race based, (**C**) Different patients gender based, and (**D**) Different age groups based. A p-value of < 0.05 was selected as a cutoff criterion.

**Figure 6:**
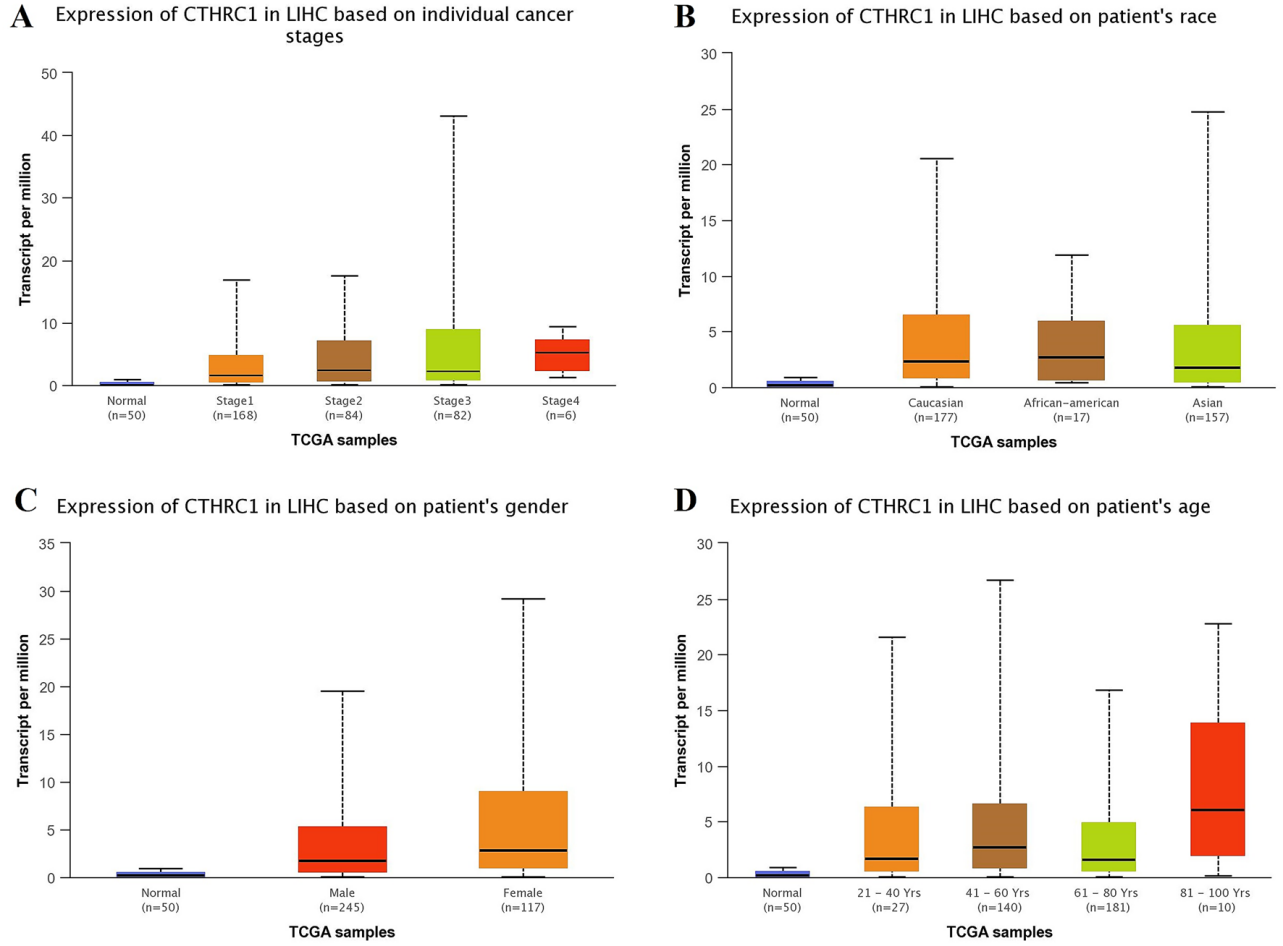
CTHRC1 transcription expression in various clinicopathological parameters of LIHC. (**A**) Different cancer stages based, (**B**) Different patients race based, (**C**) Different patients gender based, and (**D**) Different age groups based. A p-value of < 0.05 was selected as a cutoff criterion.

**Figure 7:**
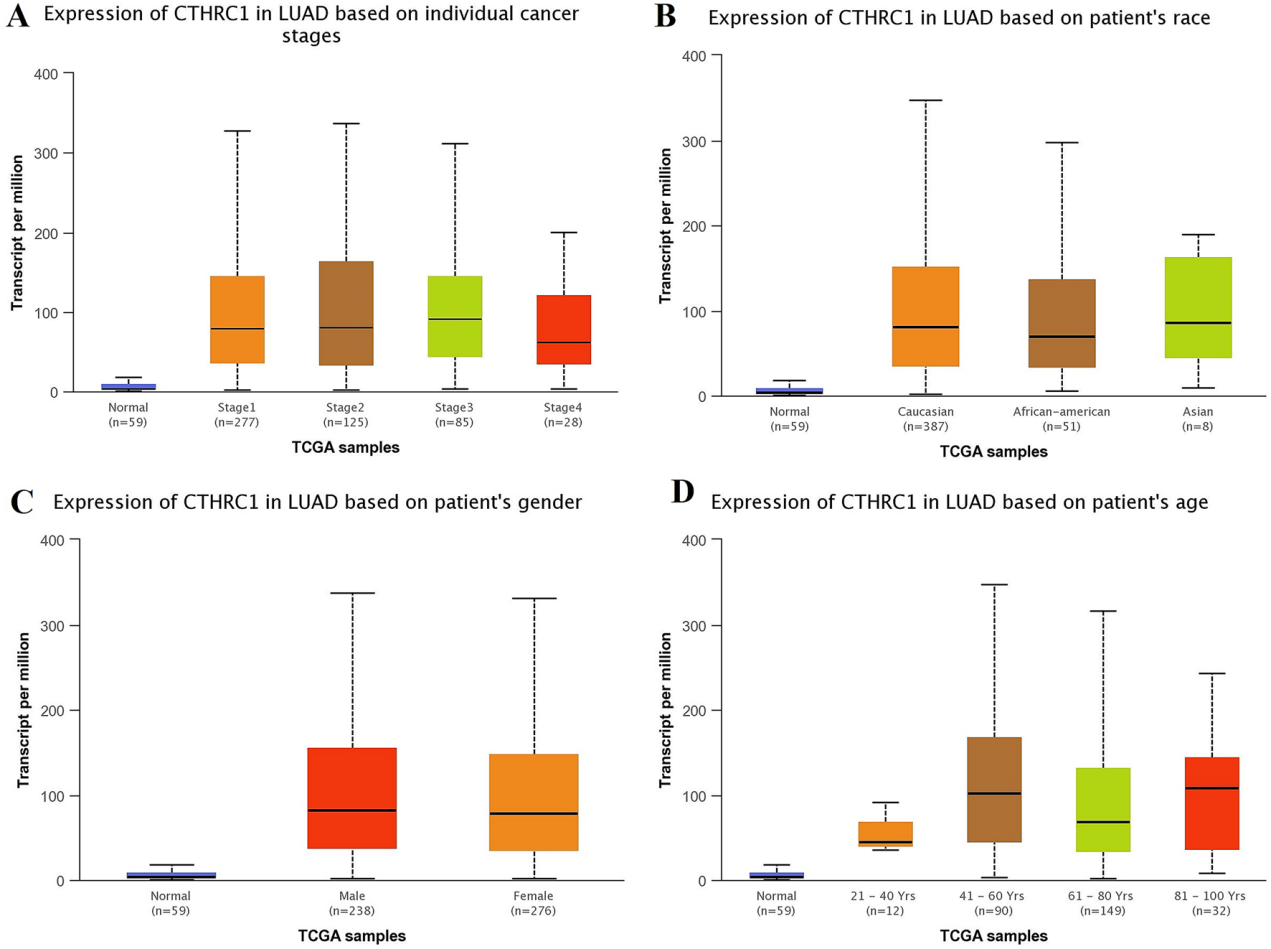
CTHRC1 transcription in various clinicopathological parameters of LUAD. (**A**) Different cancer stages based, (**B**) Different patients race based, (**C**) Different patients gender based, and (**D**) Different age groups based. A p-value of < 0.05 was selected as a cutoff criterion.

**Figure 8:**
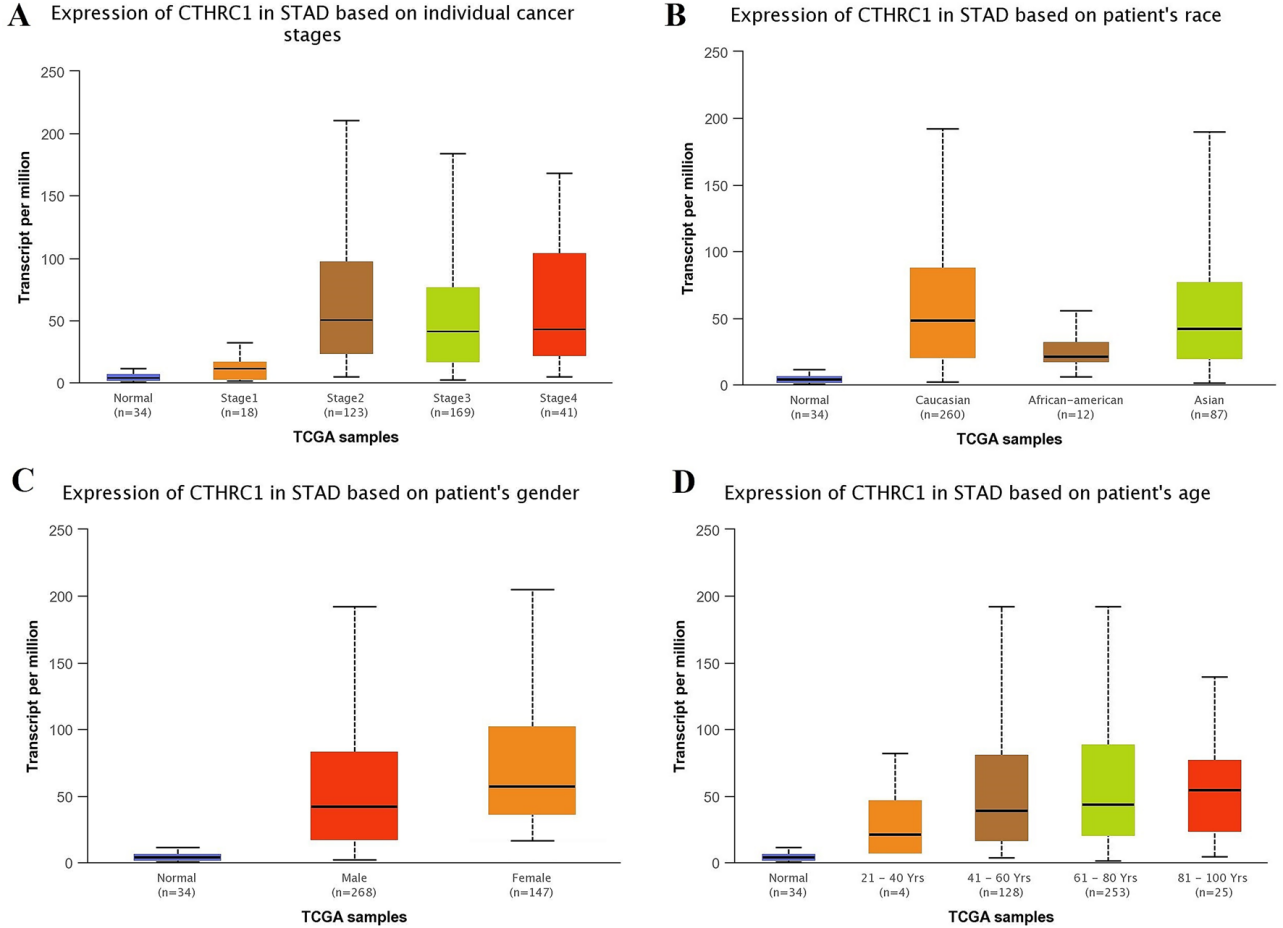
CTHRC1 transcription in various clinicopathological parameters of STAD. (**A**) Different cancer stages based, (**B**) Different patients race based, (**C**) Different patients gender based, and (**D**) Different age groups based. A p-value of < 0.05 was selected as a cutoff criterion.

**Figure 9:**
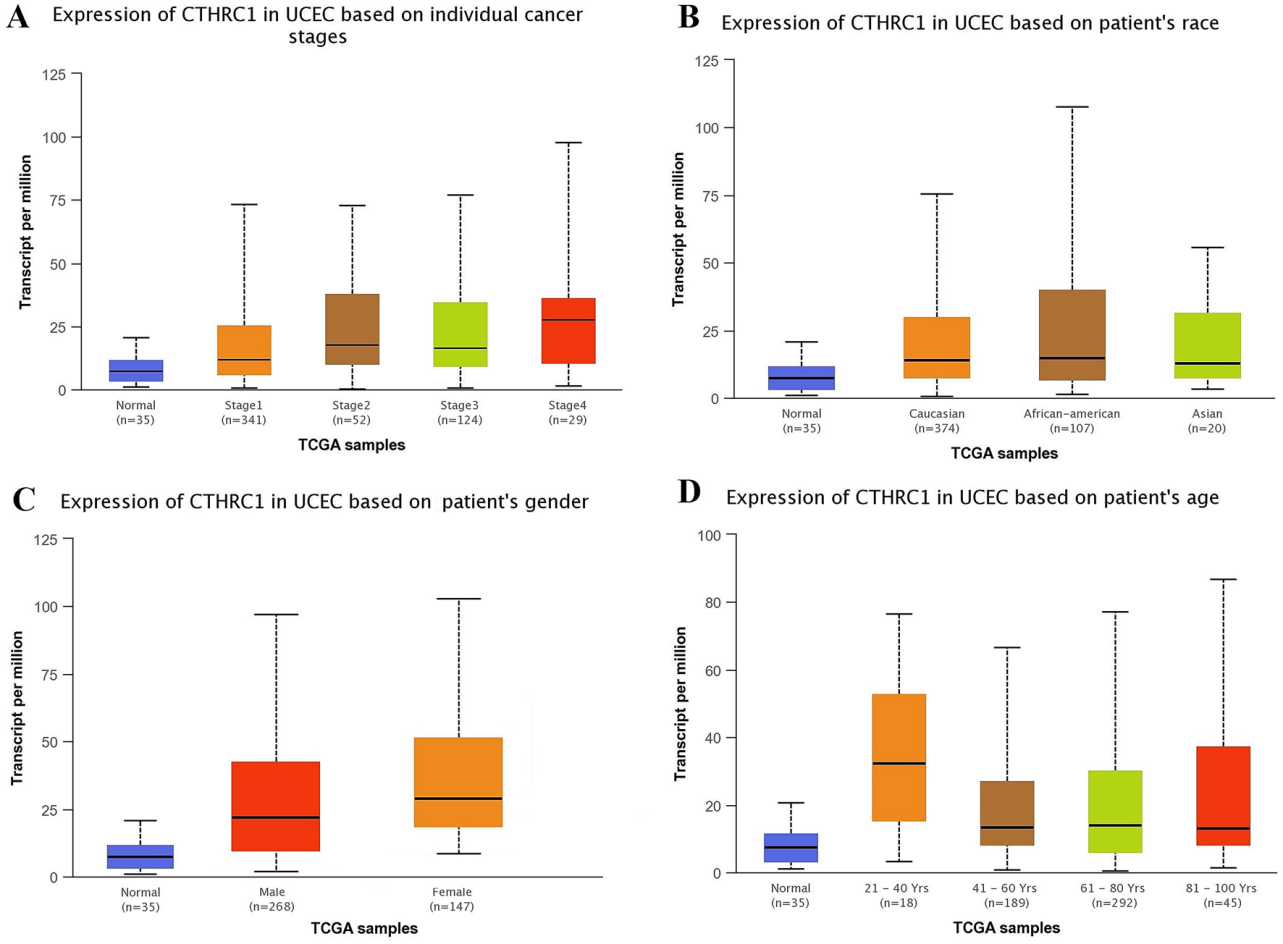
CTHRC1 transcription in various clinicopathological parameters of UCEC. (**A**) Different cancer stages based, (**B**) Different patients race based, (**C**) Different patients gender based, and (**D**) Different age groups based. A p-value of < 0.05 was selected as a cutoff criterion.

Using UALCAN and KM plotter tool, transcriptional expression analysis of CTHRC1 and its correlation analysis with the OS duration of the cancer patients showed that CTHRC1 level was significantly (p > 0.05) elevated and associated with the reduced OS duration of the HNSC, KIRC, LIHC, LUAD, STAD, and UCEC patients. Therefore, we next also explored the correlation between CTHRC1 expression level and different clinicopathological parameters of HNSC, KIRC, LIHC, LUAD, STAD, and UCEC patients. Results of the analysis revealed that CTHRC1 also significantly (p > 0.05) overexpressed in HNSC, KIRC, LIHC, LUAD, STAD, and UCEC patients of different clinicopathological characteristics stratified by cancer staging (stage 1, 2, 3, and 4), race grouping (Caucasian, African-American, and Asian), gender grouping (male and female), and age grouping (20–40 years, 41–60 years, 61–80 years, and 81–100 years) (Figs. 4, 5, 6, 7, 8, 9). Clinicopathological features based distribution of the HNSC, KIRC, LIHC, LUAD, STAD, and UCEC cohorts are given in Table 1.

**Table 1:** Clinicopathological features based distribution of the HNSC, KIRC, LIHC, LUAD, STAD, and UCEC cohorts included in the present study.

Cancer subtype	Total sample count	Types of the clinicopathological feature			
		Cancer stages based distribution	Patients ages based distribution	Patients genders based distribution	Geographical distribution
HNSC	520	Stage 1 = 27	21–40 years = 20	Male = 383	Caucasian = 444
		Stage 2 = 71	41–60 years = 236	Female = 136	African–American = 47
		Stage 3 = 81	61–80 years = 237		Asian = 11
		Stage 4 = 264	81–100 years = 24		
		Sample count with missing cancer stages data = 77	Sample count with missing patients ages data = 3	Sample count with missing patients genders data = 01	Sample count with missing geographical data = 02
		Final sample count undertaken in the analysis = 443	Final sample count undertaken in the analysis = 517	Final sample count undertaken in the analysis = 519	Final sample count undertaken in the analysis = 518
KIRC	533	Stage 1 = 267	21–40 years = 26	Male = 345	Caucasian = 462
		Stage 2 = 57	41–60 years = 238	Female = 148	African–American = 56
		Stage 3 = 123	61–80 years = 246		Asian = 08
		Stage 4 = 84	81–100 years = 23		
		Sample count with missing cancer stages data = 02	Sample count with missing patients ages data = 0	Sample count with missing patients genders data = 0	Sample count with missing geographical data = 07
		Final sample count under taken in the analysis = 531	Final sample count under taken in the analysis = 533	Final sample count undertaken in the analysis = 533	Final sample count undertaken in the analysis = 527
LIHC	371	Stage 1 = 168	21–40 years = 27	Male = 245	Caucasian = 177
		Stage 2 = 84	41–60 years = 140	Female = 117	African–American = 17
		Stage 3 = 82	61–80 years = 181		Asian = 157
		Stage 4 = 06	81–100 years = 10		
		Sample count with missing cancer stages data = 31	Sample count with missing patients ages data = 13	Sample count with missing patients genders data = 09	Sample count with missing geographical data = 20
		Final sample count under taken in the analysis = 340	Final sample count under taken in the analysis = 358	Final sample count undertaken in the analysis = 262	Final sample count undertaken in the analysis = 351
LUAD	515	Stage 1 = 277	21–40 years = 12	Male = 238	Caucasian = 387
		Stage 2 = 125	41–60 years = 90	Female = 276	African–American = 51
		Stage 3 = 85	61–80 years = 149		Asian = 08
		Stage 4 = 28	81–100 years = 32		
		Sample count with missing cancer stages data = 0	Sample count with missing patients ages data = 232	Sample count with missing patients genders data = 01	Sample count with missing geographical data = 69
		Final sample count under taken in the analysis = 515	Final sample count under taken in the analysis = 283	Final sample count undertaken in the analysis = 514	Final sample count undertaken in the analysis = 446
STAD	415	Stage 1 = 18	21–40 years = 04	Male = 268	Caucasian = 260
		Stage 2 = 123	41–60 years = 128	Female = 147	African–American = 12
		Stage 3 = 169	61–80 years = 253		Asian = 87
		Stage 4 = 41	81–100 years = 25		
		Sample count with missing cancer stages data = 64	Sample count with missing patients ages data = 05	Sample count with missing patients genders data = 0	Sample count with missing geographical data = 56
		Final sample count under taken in the analysis = 351	Final sample count under taken in the analysis = 410	Final sample count undertaken in the analysis = 415	Final sample count undertaken in the analysis = 359
UCEC	546	Stage 1 = 341	21–40 years = 18	Male = 268	Caucasian = 374
		Stage 2 = 52	41–60 years = 189	Female = 147	African–American = 107
		Stage 3 = 124	61–80 years = 292		Asian = 20
		Stage 4 = 29	81–100 years = 45		
		Sample count with missing cancer stages data = 0	Sample count with missing patients ages data = 02	Sample count with missing patients genders data = 131	Sample count with missing geographical data = 45
		Final sample count under taken in the analysis = 546	Final sample count under taken in the analysis = 544	Final sample count undertaken in the analysis = 415	Final sample count undertaken in the analysis = 501

### Transcription expression level validation of CTHRC1 using independent HNSC, KIRC, LIHC, LUAD, STAD, and UCEC cohorts

**Figure 10:**
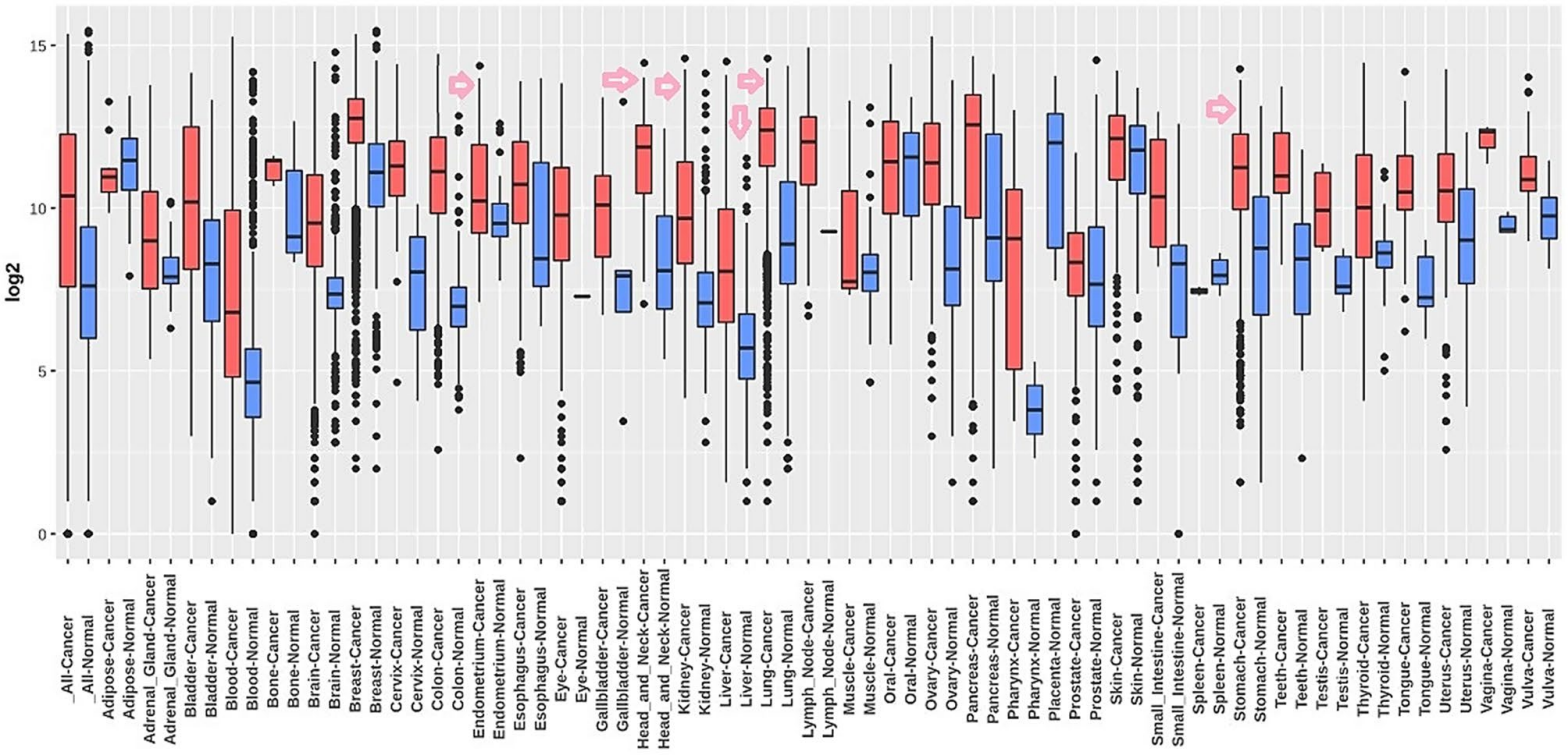
Transcription expression level validation of CTHRC1 using independent HNSC, KIRC, LIHC, LUAD, STAD, and UCEC cohorts via GENT2 database. A p-value of < 0.05 was selected as a cutoff criterion.

To further validate the transcription expression level of CTHRC1, we re-analyze its expression using independent cohorts of HNSC, KIRC, LIHC, LUAD, STAD, and UCEC from Affymetrix U133A and U133Plus2 microarray platforms via GENT2 platform. The results of re-analysis also revealed its significant (p > 0.05) overexpression in HNSC, KIRC, LIHC, LUAD, STAD, and UCEC patients relative to normal controls (Fig. 10). Information of the HNSC, KIRC, LIHC, LUAD, STAD, and UCEC datasets used for CTHRC1 expression validation is given in Table 2.

**Table 2:** Information of the HNSC, KIRC, LIHC, LUAD, STAD, and UCEC datasets used for the CTHRC1 expression validation via GENT2 webserver.

Sr. no.	Cancer	Datasets	Source
1	HNSC	GSE6791, GSE10300, GSE29330, GSE3292, GSE31287, GSE6791, and GSE29330	Affymetrix U133A and U133 Plus2 microarray platforms
2	KIRC	GSE2109, GSE46699, GSE47352, GSE53224, GSE53757, GSE7023, GSE68629, GSE7392, GSE8271, GSE11045, GSE11151, GSE12090, GSE12606, GSE14762, GSE19982, GSE22541, GSE36895, GSE53757, and GSE11151	
3	LIHC	GSE45436, GSE49515, GSE2109, GSE58208, GSE6222, GSE62232, GSE6764, GSE75285, GSE9843, GSE40367, and GSE40873, GSE41804	
4	LUAD	GSE40791, GSE37745, GSE2109, GSE43346, GSE43580, GSE50081, GSE30219, GSE63074, GSE64766, GSE77803, GSE10445, GSE19188, GSE27262, GSE33532, GSE40791, GSE5058, and GSE7307	
5	STAD	GSE42252, GSE49515, GSE51105, GSE51725, GSE57308, GSE66229, GSE64951, GSE79973, GSE13911, GSE15459, GSE17187, GSE22377, GSE34942, GSE35809, GSE43346, GSE79973, and GSE13911	
6	UCEC	GSE2109, GSE19959, GSE4888, GSE6364, GSE7307, and GSE7307	

### Translational expression level of CTHRC1 in head and neck, kidney, liver, lung, stomach, and endometrial cancers

**Figure 11:**
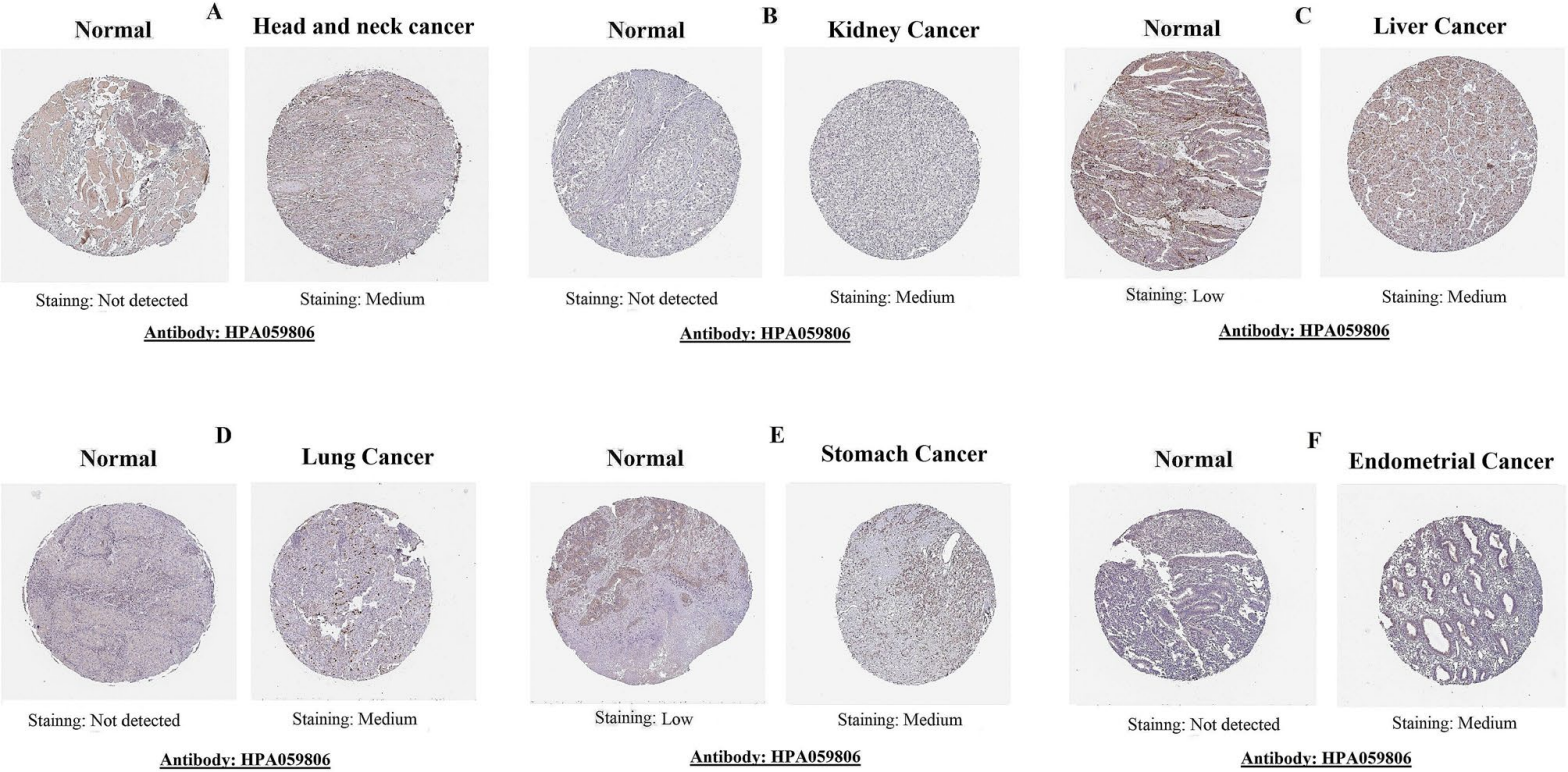
Translation expression of CTHRC1 across distinct cancer subtypes paired with normal controls taken from Human Protein Atlas (HPA) database (× 200). (**A**) Head and neck cancer, (**B**) kidney cancer, (**C**) liver cancer, (**D**) lung cancer, (**E**) stomach cancer, and (**F**) endometrial cancer.

We also analyzed the CTHRC1 translational level in normal and head and neck, kidney, liver, lung, stomach, and endometrial cancers tissues using HPA. The obtained images from HPA revealed that CTHRC1 protein was not expressed or detected at a low level in head and neck (not expressed), kidney (not expressed), liver (low), lung (not expressed), and stomach (low), and endometrial normal tissues. However, its overexpression (medium) was detected in cancer tissues of the head and neck, kidney, liver, lung, stomach, and endometrial (Fig. 11).

### CTHRC1 promoter methylation, genetic alterations, and CNVs analysis in HNSC, KIRC, LIHC, LUAD, STAD, and UCEC

**Figure 12:**
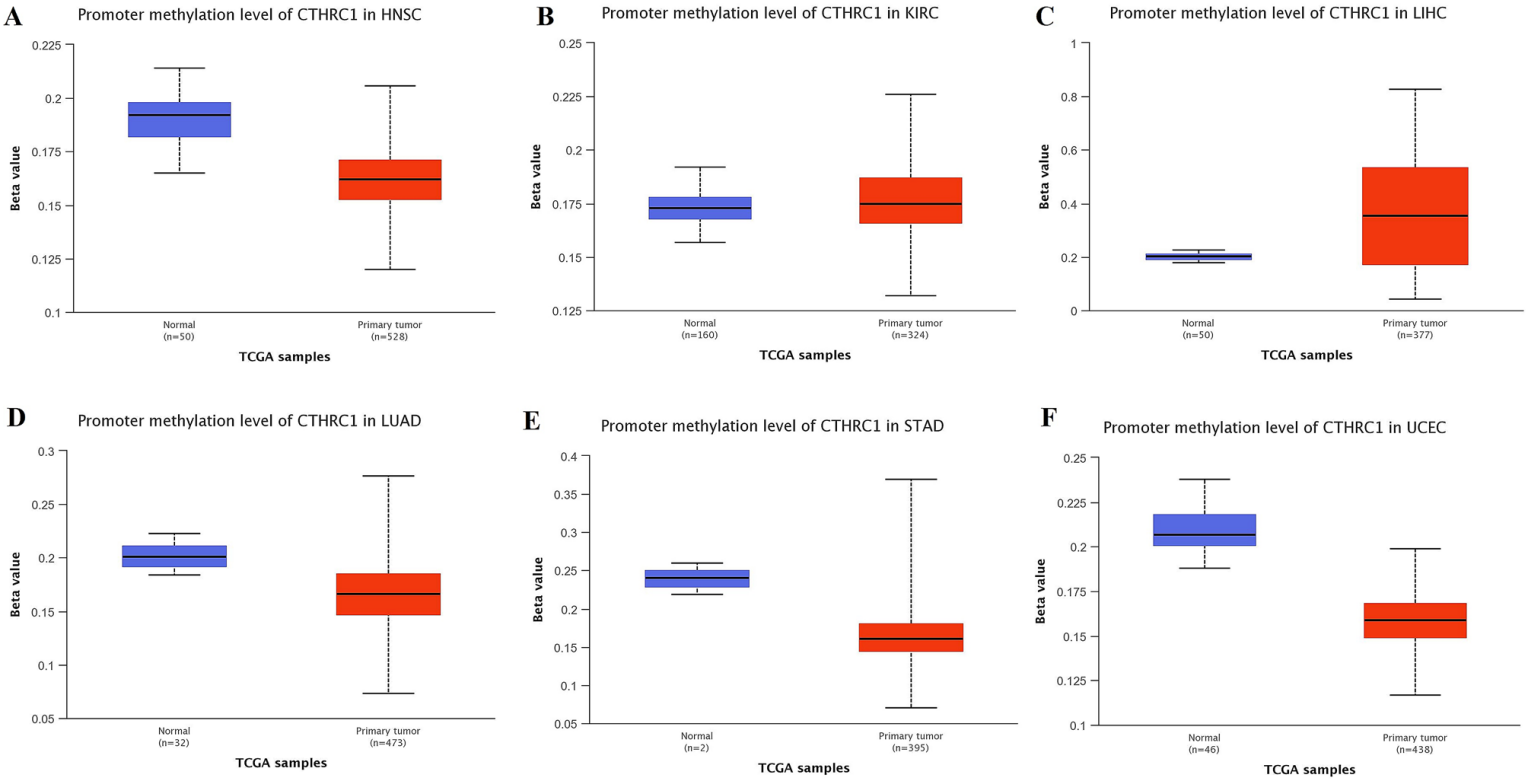
Promoter methylation level of CTHEC1 in HNSC, KIRC, LIHC, LUAD, STAD, and UCEC. (**A**) HNSC, (**B**) KIRC, (**C**) LIHC, (**D**) LUAD, (**E**) STAD, and, (**F**) UCEC. A p-value of < 0.05 was selected as a cutoff criterion.

Promoter methylation is a key epigenetic mechanism that regulates transcription gene expression and plays a vital role in tumorgenesis^27^. Therefore, we further analyzed that whether CTHRC1 transcription expression level was influenced by its promoter methylation level or not using UALCAN. As highlighted in Fig. 12, the box plots indicated that CTHRC1 transcription expression level was significantly (p > 0.005) influenced by its promoter hypomethylation in HNSC and UCEC, therefore, we speculate that the up-regulation of CTHRC1 in HNSC and UCEC might be the outcome of its promoter hypomethylation. However, the observed significant hypermethylation of CTHRC1 promoter in KIRC, LIHC, LUAD, and STAD challenges the classical view of methylation where overexpression is always associated with hypomethylation. Therefore, further detailed work is required to be done to explore the connection between hypermethylation of CTHRC1 promoter and its expression in KIRC, LIHC, LUAD, and STAD.

**Figure 13:**
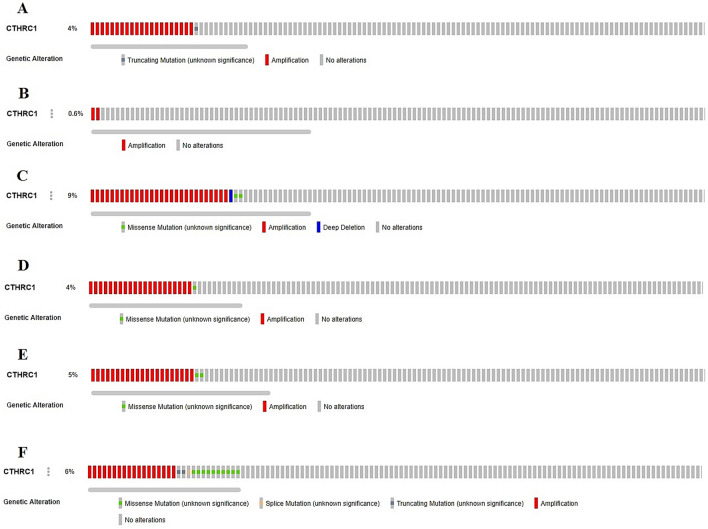
CTHRC1 genetic alterations and copy number variations (CNVs) in TCGA HNSC, KIRC, LIHC, LUAD, STAD, and UCEC datasets. (**A**) HNSC, (**B**) KIRC, (**C**) LIHC, (**D**) LUAD, (**E**) STAD, and, (**F**) UCEC.

In addition to promoter methylation, we also analyzed the contribution of genetic alterations and CNVs in the up-regulation of CTHRC1 via cBioPortal webserver using PanCancer Atlas datasets of HNSC, KIRC, LIHC, LUAD, STAD, and UCEC from TCGA. Our results revealed that CTHRC1 was genetically altered in only 4%, 0.6%, 9%, 4%, 5%, and 6% of the queued HNSC, KIRC, LIHC, LUAD, STAD, and UCEC samples, respectively, and deep amplification genetic abnormality was most frequent in these cancer subtypes (Fig. 13). Altogether, these results suggested that deep amplification may also participate in the overexpression of CTHRC1 in HNSC, KIRC, LIHC, LUAD, STAD, and UCEC.

### Pathway enrichment analysis of CTHRC1

**Figure 14:**
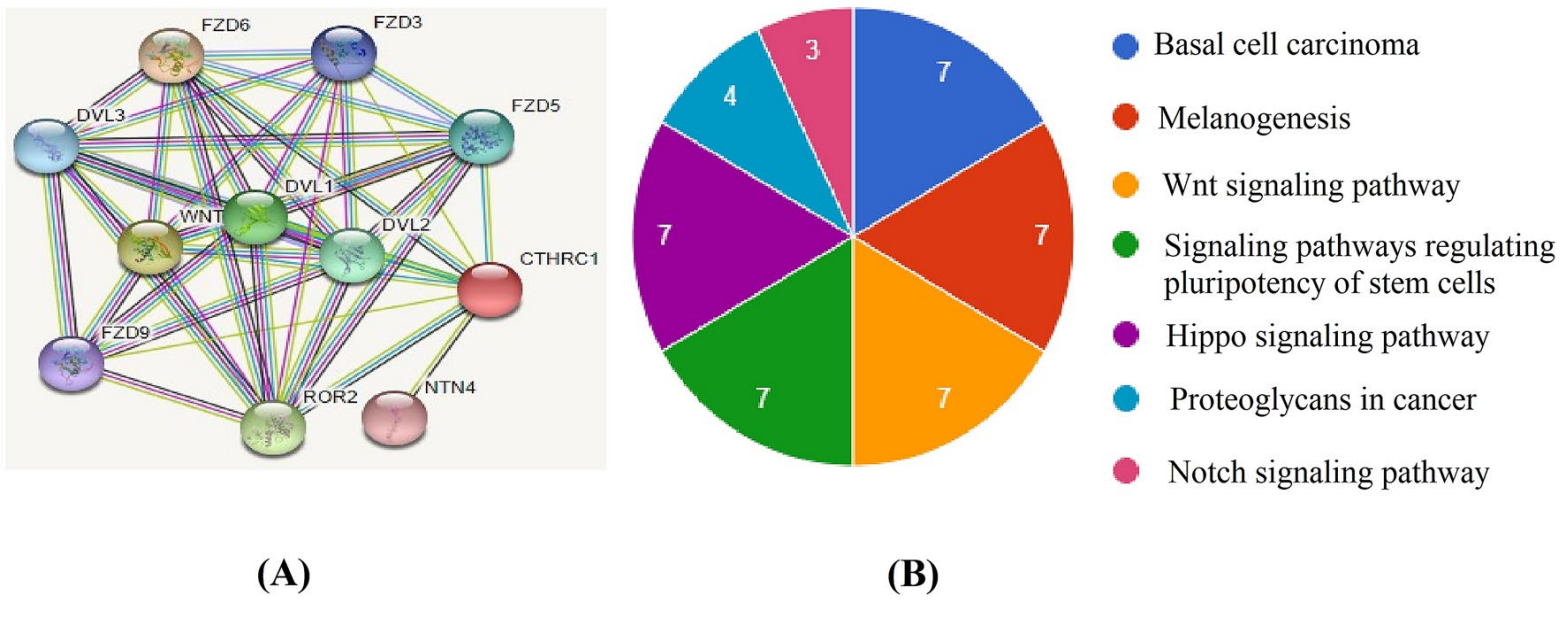
PPI network and Kyoto encyclopedia of genes and genomes (KEGG) pathway analysis of the CTHRC1 enriched genes. (**A**) A PPI network of CTHRC1 enriched genes, (**B**) KEGG pathway analysis of the CTHRC1 enriched genes.

To explore the CTHRC1 enriched pathways, a protein–protein interaction (PPI) network of CTHRC1 associated genes was obtained using the STRING database and visualized through Cytoscape. In total 11 nodes and 138 edges were found in the obtained PPI network (Fig. 13A). Using David tool, we revealed that CTHRC1-related genes were involved in seven different pathways including Basal cell carcinoma, Melanogenesis, Wnt signaling pathway, Signaling pathways regulating pluripotency of stem cells, Hippo signaling pathway, Proteoglycans in cancer, and Notch signaling pathway (Fig. 14; Table 3).

**Table 3:** Detail of Kyoto encyclopedia of genes and genomes pathway analysis of the CTHRC1 enriched genes.

Pathway	Description	Gene count	Enriched genes	p-value
hsa05217	Basal cell carcinoma	7	FZD3, FZD5, FZD6, DVL1, DVL2, FZD9, DVL3	< 0.05
hsa04916	Melanogenesis	7	FZD3, FZD5, FZD6, DVL1, DVL2, FZD9, DVL3	< 0.05
hsa04310	Wnt signaling pathway	7	FZD3, FZD5, FZD6, DVL1, DVL2, FZD9, DVL3	< 0.05
hsa04550	Signaling pathways regulating pluripotency of stem cells	7	FZD3, FZD5, FZD6, DVL1, DVL2, FZD9, DVL3	< 0.05
hsa04390	Hippo signaling pathway	7	FZD3, FZD5, FZD6, DVL1, DVL2, FZD9, DVL3	< 0.05
hsa05205	Proteoglycans in cancer	4	FZD3, FZD5, FZD6, FZD9	< 0.05
hsa04330	Notch signaling pathway	3	DVL1, DVL2, DVL3	< 0.05

### CD8+ T immune cells infiltration and tumor purity of CTHRC1 in HNSC, KIRC, LIHC, LUAD, STAD, and UCEC patients

**Figure 15:**
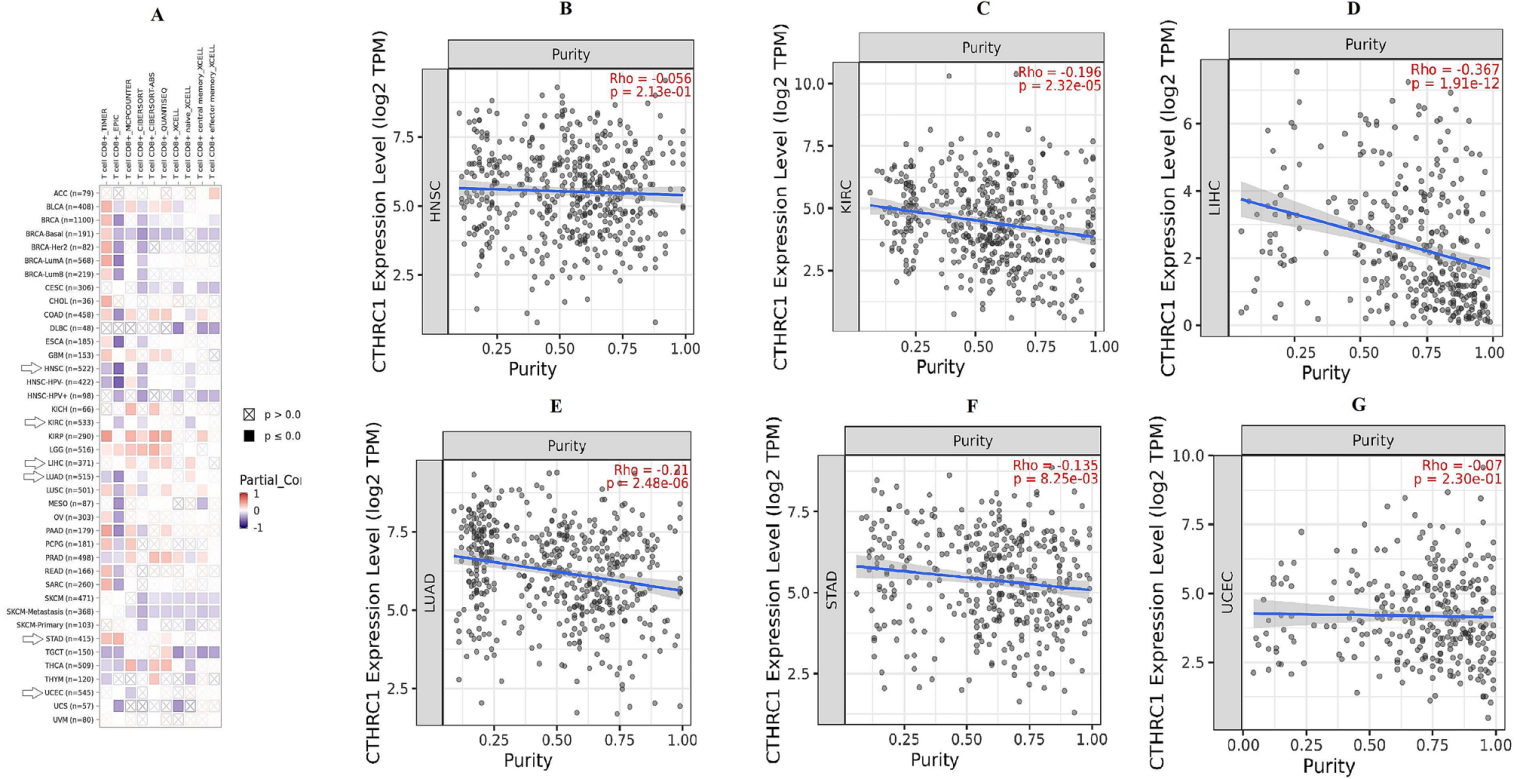
TIMER based Spearman correlational analysis between CTHRC1 expression and CD8+ T immune infiltration and tumor purity in HNSC, KIRC, LIHC, LUAD, STAD, and UCEC. (**A**) A correlation analysis between CTHRC1 expression and CD8+ T immune infiltration in HNSC, KIRC, LIHC, LUAD, STAD, and UCEC, (**B**) a correlation analysis between CTHRC1 expression and tumor purity in HNSC, (**C**) a correlation analysis between CTHRC1 expression and tumor purity in KIRC, (**D**) a correlation analysis between CTHRC1 expression and tumor purity in LIHC, (**E**) a correlation analysis between CTHRC1 expression and tumor purity in LUAD, (**F**) a correlation analysis between CTHRC1 expression and tumor purity in STAD, and (**G**) a correlation analysis between CTHRC1 expression and tumor purity in UCEC.

Tumor development is closely associated with immunity^28^. As CD8+ T immune cells are the major components of the immune system, we evaluated the Spearman correlation between CTHRC1 expression and CD8+ T immune infiltration in HNSC, KIRC, LIHC, LUAD, STAD, and UCEC via TIMER database. Results of this analysis suggested a significant (p > 0.005) negative correlation between CD8+ T immune cells level and CTHRC1 higher expression in HNSC, KIRC, LUAD, and UCEC while a significant (p > 0.005) positive correlation between these two parameters in LIHC and STAD (Fig. 15). Collectivly these results highlighted a significant relationship between the CTHRC1 expression and CD8+ T immune cells infiltration. In addition, we have also explored the correlation between CTHRC1 expression and tumor purity in HNSC, KIRC, LIHC, LUAD, STAD, and UCEC using TIMER. Analysis results showed that CTHRC1 expression level was negatively related to the tumor purity in HNSC (Rho = − 0.05, p = 2.13e − 01), KIRC (Rho = − 0.192, p = 2.32e − 05), LIHC (Rho = − 0.367, p = 1.91e − 12), LUAD (Rho = − 0.21, p = 2.48e − 06), STAD (Rho = − 0.135, p = 8.25e − 03), and UCEC (Rho = − 0.07, p = 2.30e − 01) (Fig. 15).

### Gene–drug interaction network analysis of the CTHRC1

**Figure 16:**
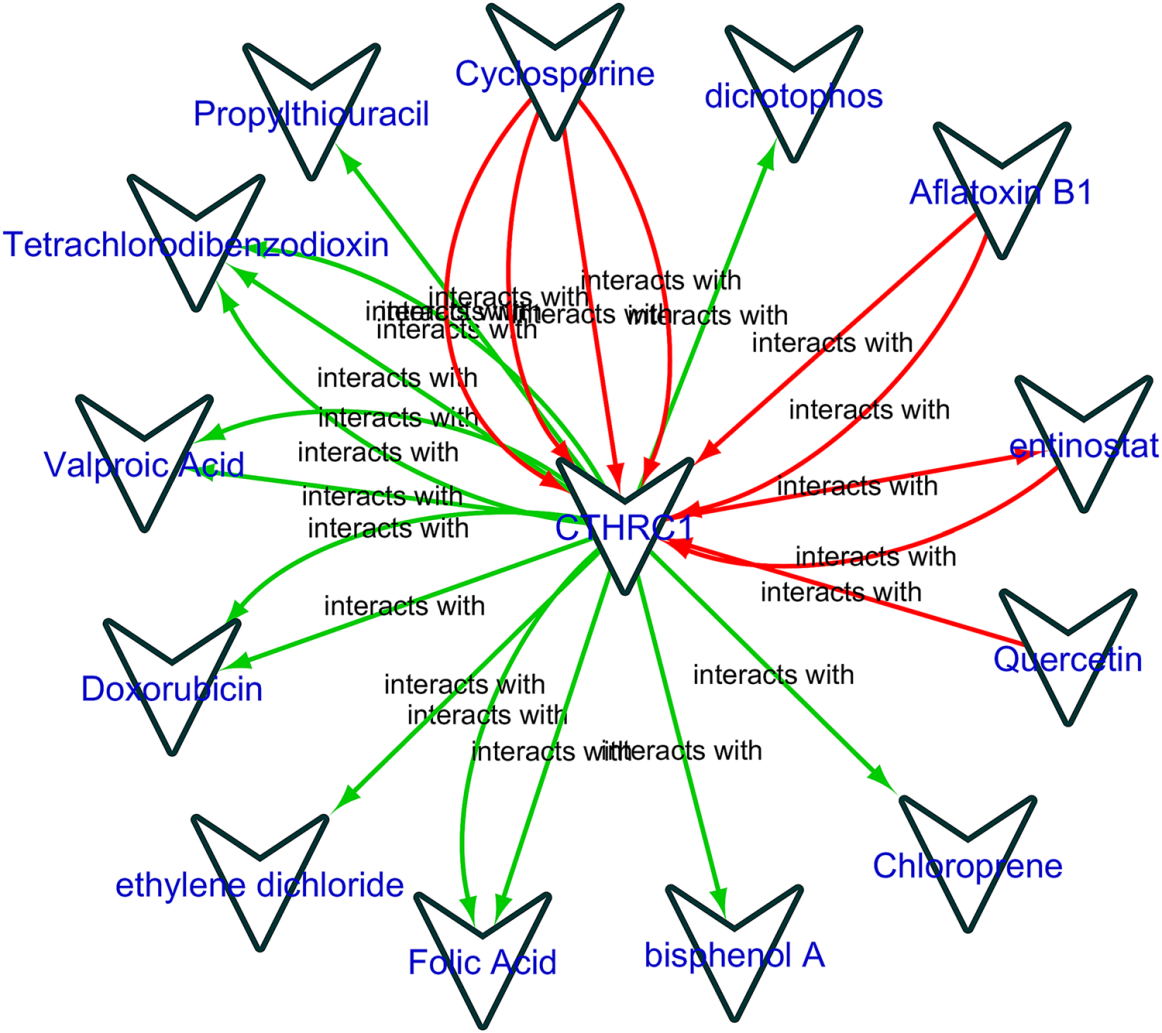
Gene–drug interaction network of the CTHRC1 and chemotherapeutic drugs. Red arrows: chemotherapeutic drugs increase the expression of CTHRC1; green arrows: chemotherapeutic drugs decrease the expression of CTHRC1. The numbers of arrows between chemotherapeutic drugs and key genes in this network represent the supported numbers of literatures by previous reports.

In order to explore the relationship between CTHRC1 and available cancer therapeutic drugs, a gene–drug interaction network was developed using the CTD database. The expression of CTHRC1 could potentially influence by a variety of drugs. For example, cyciosperine and dicrotophos could elevate the expression level of CTHRC1 while valporic acid and doxorubicin could reduce CTHRC1 expression level (Fig. 16).

## Discussion

CTHRC1 is an extracellular matrix protein that has earlier been linked to different cancers including cervical carcinoma^6^, non-small cell lung cancer^29^, endometrial cancer^8^, colorectal, breast, and prostate cancer^9–11^ by the limited number of studies. In the current study, we comprehensively analyzed CTHRC1 in distinct human cancer subtypes for the first time and find more closely CTHRC1-associated cancer subtypes through the integrated analyses approach.

In our study, we revealed that CTHRC1 expression was elevated in 24 major subtypes of human cancers, compared to normal controls. Moreover, CTHRC1 elevated expression was significantly associated with the reduced OS duration of the HNSC, KIRC, LIHC, LUAD, STAD, and UCEC, these results suggested the unique role of CTHRC1 up-regulation in the tumorgenesis of HNSC, KIRC, LIHC, LUAD, STAD, and UCEC. Therefore, in the present study, our main focus is these six cancers.

Following this, we next re-analyzed the CTHRC1 expression in different clinicopathological features of HNSC, KIRC, LIHC, LUAD, STAD, and UCEC, as a results, we also observed the significant (p < 0.05) up-regulation of CTHRC1 in different clinicopathological features of HNSC, KIRC, LIHC, LUAD, STAD, and UCEC including different cancer stages, patients races, genders, and age groups.

Epigenetic alterations are the main causes of gene expression fluctuations^30^. CTHRC1 has been reported to be overexpressed by its promoter hypomethylation in gastric cancer by earlier research^31^. In this study, we have shown that CTHRC1 expression was significantly affected by its promoter hypomethylation in HNSC and UCEC. Beside, along with up-regulation, we also revealed the hypermethylation of CTHRC1 promoter in KIRC, LIHC, LUAD, and STAD. This scenario challenges the classical view of methylation where overexpression is always associated with hypomethylation and demands further extensive research to resolve the conflict. Furthermore, genetic mutations and CNVs, including deep amplification, gain, loss, and deep deletion are known to alter the gene expression in tumorgenesis^32^. In our study, we observed that CTHRC1 was genetically altered in only 4%, 0.6%, 9%, 4%, 5%, and 6% of the queued HNSC, KIRC, LIHC, LUAD, STAD, and UCEC samples, respectively. The most frequently observed genetic abnormality in these cancer was maximum deep amplification. Taken together, our data suggest the slight influence of CNVs on the up-regulation of CTHRC1 in HNSC, KIRC, LIHC, LUAD, STAD, and UCEC.

Although recent studies have explored several expression based diagnostic and prognostic biomarkers in HNSC, KIRC, LIHC, LUAD, STAD, and UCEC, such as EGFR^33,34^, CCND1^35^, ERCC1^36^, p16^37^, Bcl-2^37^, FGFR^37^, CCND^38^, and KLK-6^39^ in HNSC, ACAA1, ALDH6A1, AUH, ACADSB, HADH, ACAA1, and PCCA in KIRC^40^, TP53, TTN, CTNNB1, MUC16, PCLO, CDKN2A, ALDH6A1, and LPCAT1 in LIHC^41^, PECAM1, CDK1, MKI67, SPP1, TOP2A, CHEK1, CCNB1, and RRM2 in LUAD^42^, KLF4, CGN, LIF, SHH, GATA6, FOXA2, OCLN, FOXA1, CLDN1, and NQO1 in STAD^43^, and BUB1, TOP2A, CDCA8, TTK, ASPM, UBE2C, BIRC5, HJURP, CENPA, MCM10, FOXM1, SPAG5, EXO1, ESPL1, OIP5, MCM4, CDC25C, DEPDC1, KIF18B, ERCC6L in UCEC^44^. However, best to our knowledge, none of these or any other biomarkers have been generalized so far in HNSC, KIRC, LIHC, LUAD, STAD, and UCEC patients of different clinicopathological features. In our study, we have shown the significant (p < 0.05) up-regulation of CTHRC1 expression in HNSC, KIRC, LIHC, LUAD, STAD, and UCEC patients of various clinicopathological features (different cancer stages, patients races, genders, and age groups) as compared to the normal controls. Furthermore, CTHRC1 promoter methylation level and OS information have also proven its useful values as a novel potential biomarker of these cancers. Therefore, this is the first study that reported the shared clinicopathological features-specific diagnostic and prognostic potential of CTHRC1 in six different cancers including HNSC, KIRC, LIHC, LUAD, STAD, and UCEC, which may provide new therapeutic possibilities for cancer patients.

Immunotherapy has proven to be very successful in treating solid tumors^45^. Interestingly, we revealed that there is a close correlation between CTHRC1 expression and CD8+ T immune infiltration, which may contribute to the development of HNSC, KIRC, LIHC, LUAD, STAD, and UCEC by affecting the tumor microenvironment. To date, no previous study has reported these such correlations between CTHRC1 and CD8+ T cells level in HNSC, KIRC, LIHC, LUAD, STAD, and UCEC patients of different clinicopathological features. Therefore, this valuable knowledge may help to design more precise immunotherapy for cancer patients. Moreover, we have also found that CTHRC1 expression was inversely correlated with tumor purity in HNSC, KIRC, LIHC, LUAD, STAD, and UCEC, this knowledge may also help in predicting the clinical outcomes in HNSC, KIRC, LIHC, LUAD, STAD, and UCEC.

Protein–protein interactions are the core of biological pathways. The CTHRC1 PPI network revealed a set of ten genes that directly interact with CTHRC1. Most of them were involved in different signaling pathways including Basal cell carcinoma, Melanogenesis, Wnt signaling pathway, Signaling pathways regulating pluripotency of stem cells, Hippo signaling pathway, Proteoglycans in cancer, and Notch signaling pathway. Additionally, we have also identified few potential drugs that could influence the expression level of CTHRC1 in cancer, however, whether HNSC, KIRC, LIHC, LUAD, STAD, and UCEC patients with overexpressed CTHRC1 can benefit from these drugs or whether CTHRC1 may be targeted by these drugs in the treatment of these cancers needs biological experimental support.

## Conclusion

In conclusion, we indicated that CTHRC1 was overexpressed in HNSC, KIRC, LIHC, LUAD, STAD, and UCEC tissues relative to normal tissues. Additionally, CTHRC1 overexpression was significantly associated with different clinicopathological features, reduced OS duration, and CD8+ T immune infiltration and tumor purity. In summary, our research is a preliminary study that reported the shared clinicopathological features-specific diagnostic and prognostic potential of CTHRC1 in six different cancers including HNSC, KIRC, LIHC, LUAD, STAD, and UCEC.

## References

[1] Ma X, Yu H (2006). Global burden of cancer. Yale J. Biol. Med..

[2] Siegel RL, Miller KD, Jemal A (2020). Cancer statistics, 2020. CA Cancer J. Clin..

[3] Srigley JR, Delahunt B, Eble JN, Egevad L, Epstein JI (2013). The International Society of Urological Pathology (ISUP) vancouver classification of renal neoplasia. Am. J. Surg. Pathol..

[4] Siegel R, Ward E, Brawley O, Jemal A (2011). Cancer statistics, 2011: The impact of eliminating socioeconomic and racial disparities on premature cancer deaths. CA Cancer J. Clin..

[5] Liu W, Fu X-L, Yang J-Y, Yang M-W, Tao L-Y (2016). Elevated expression of CTHRC1 predicts unfavorable prognosis in patients with pancreatic ductal adenocarcinoma. Am. J. Cancer Res..

[6] Xu G, Fan W, Wang F, Lu H, Xing X (2018). CTHRC1 as a novel biomarker in the diagnosis of cervical squamous cell carcinoma. Int. J. Clin. Exp. Pathol..

[7] He W, Zhang H, Wang Y, Zhou Y, Luo Y (2018). CTHRC1 induces non-small cell lung cancer (NSCLC) invasion through upregulating MMP-7/MMP-9. BMC Cancer.

[8] Li L-Y, Yin K-M, Bai Y-H, Zhang Z-G, Di W (2019). CTHRC1 promotes M2-like macrophage recruitment and myometrial invasion in endometrial carcinoma by integrin-Akt signaling pathway. Clin. Exp. Metas..

[9] Ma Z, Chao F, Wang S, Song Z, Zhuo Z (2020). CTHRC1 affects malignant tumor cell behavior and is regulated by miR-30e-5p in human prostate cancer. Biochem. Biophys. Res. Commun..

[10] Chen Y, Sun Y, Cui Y, Lei Y, Jiang N (2019). High CTHRC1 expression may be closely associated with angiogenesis and indicates poor prognosis in lung adenocarcinoma patients. Cancer Cell Int..

[11] Ni S, Ren F, Xu M, Tan C, Weng W (2018). CTHRC1 overexpression predicts poor survival and enhances epithelial–mesenchymal transition in colorectal cancer. Cancer Med..

[12] Chandrashekar DS, Bashel B, Balasubramanya SAH, Creighton CJ, Ponce-Rodriguez I (2017). UALCAN: A portal for facilitating tumor subgroup gene expression and survival analyses. Neoplasia (New York, NY).

[13] Maciejczyk A, Szelachowska J, Czapiga B, Matkowski R, Hałoń A (2013). Elevated BUBR1 expression is associated with poor survival in early breast cancer patients: 15-year follow-up analysis. J. Histochem. Cytochem..

[14] Zheng H, Zhang G, Zhang L, Wang Q, Li H (2020). Comprehensive review of web servers and bioinformatics tools for cancer prognosis analysis. Front. Oncol..

[15] Xie L, Wang Q, Dang Y, Ge L, Sun X (2019). OSkirc: A web tool for identifying prognostic biomarkers in kidney renal clear cell carcinoma. Future Oncol..

[16] An, Y. *et al*. OSlihc: An online prognostic biomarker analysis tool for hepatocellular carcinoma. *Front. Pharmacol*. **10**(11), 875 (2020). 10.3389/fphar.2020.00875 PMC7298068 32587519

[17] Yan Z, Wang Q, Lu Z, Sun X, Song P (2020). OSluca: An interactive web server to evaluate prognostic biomarkers for lung cancer. Front. Genet..

[18] Tang Z, Li C, Kang B, Gao G, Li C (2017). GEPIA: A web server for cancer and normal gene expression profiling and interactive analyses. Nucleic Acids Res..

[19] Park S-J, Yoon B-H, Kim S-K, Kim S-Y (2019). GENT2: An updated gene expression database for normal and tumor tissues. BMC Med. Genom..

[20] Uhlén, M., Fagerberg, L., Hallström, B.M., Lindskog, C., Oksvold, P., *et al*. Tissue-based map of the human proteome. *Science*. **347**(6220) (2015). 10.1126/science.1260419 25613900

[21] Cerami E, Gao J, Dogrusoz U, Gross BE, Sumer SO (2012). The cBio cancer genomics portal: An open platform for exploring multidimensional cancer genomics data. Cancer Discov..

[22] von Mering C, Huynen M, Jaeggi D, Schmidt S, Bork P (2003). STRING: A database of predicted functional associations between proteins. Nucleic Acids Res..

[23] Shannon P, Markiel A, Ozier O, Baliga NS, Wang JT (2003). Cytoscape: A software environment for integrated models of biomolecular interaction networks. Genome Res..

[24] Huang DW, Sherman BT, Tan Q, Collins JR, Alvord WG (2007). The DAVID Gene Functional Classification Tool: A novel biological module-centric algorithm to functionally analyze large gene lists. Genome Biol..

[25] Li T, Fu J, Zeng Z, Cohen D, Li J (2020). TIMER2.0 for analysis of tumor-infiltrating immune cells. Nucleic Acids Res..

[26] Mattingly CJ, Colby GT, Forrest JN, Boyer JL (2003). The Comparative Toxicogenomics Database (CTD). Environ. Health Perspect..

[27] Moore LD, Le T, Fan G (2013). DNA methylation and its basic function. Neuropsychopharmacology.

[28] Pandya PH, Murray ME, Pollok KE, Renbarger JL (2016). The immune system in cancer pathogenesis: Potential therapeutic approaches. J. Immunol. Res..

[29] Ke Z, He W, Lai Y, Guo X, Chen S (2014). Overexpression of collagen triple helix repeat containing 1 (CTHRC1) is associated with tumour aggressiveness and poor prognosis in human non-small cell lung cancer. Oncotarget.

[30] Grewal SI, Moazed D (2003). Heterochromatin and epigenetic control of gene expression. Science (New York, NY).

[31] Sun XJ, Wang MC, Zhang FH, Kong X (2018). An integrated analysis of genome-wide DNA methylation and gene expression data in hepatocellular carcinoma. FEBS Open Bio.

[32] Hudler, P. Genetic aspects of gastric cancer instability. *Sci. World J*. 2012 (2012). 10.1100/2012/761909 PMC3353315 22606061

[33] Chung CH, Ely K, McGavran L, Varella-Garcia M, Parker J (2006). Increased epidermal growth factor receptor gene copy number is associated with poor prognosis in head and neck squamous cell carcinomas. J. Clin. Oncol..

[34] Ang KK, Berkey BA, Tu X, Zhang H-Z, Katz R (2002). Impact of epidermal growth factor receptor expression on survival and pattern of relapse in patients with advanced head and neck carcinoma. Can. Res..

[35] Borchiellini D, Etienne-Grimaldi M, Bensadoun R, Benezery K, Dassonville O (2017). Candidate apoptotic and DNA repair gene approach confirms involvement of ERCC1, ERCC5, TP53 and MDM2 in radiation-induced toxicity in head and neck cancer. Oral Oncol..

[36] Prochnow S, Wilczak W, Bosch V, Clauditz T, Muenscher A (2019). ERCC1, XPF and XPA—Locoregional differences and prognostic value of DNA repair protein expression in patients with head and neck squamous cell carcinoma. Clin. Oral Invest..

[37] Rasmussen JH, Håkansson K, Rasmussen GB, Vogelius IR, Friborg J (2018). A clinical prognostic model compared to the newly adopted UICC staging in an independent validation cohort of P16 negative/positive head and neck cancer patients. Oral Oncol..

[38] Karamboulas C, Bruce JP, Hope AJ, Meens J, Huang SH (2018). Patient-derived xenografts for prognostication and personalized treatment for head and neck squamous cell carcinoma. Cell Rep..

[39] Schrader CH, Kolb M, Zaoui K, Flechtenmacher C, Grabe N (2015). Kallikrein-related peptidase 6 regulates epithelial-to-mesenchymal transition and serves as prognostic biomarker for head and neck squamous cell carcinoma patients. Mol. Cancer.

[40] Zhang B, Wu Q, Wang Z, Xu R, Hu X (2019). The promising novel biomarkers and candidate small molecule drugs in kidney renal clear cell carcinoma: Evidence from bioinformatics analysis of high-throughput data. Mol. Genet. Genom. Med..

[41] Wang, R., Hu, X., Liu, X., Bai, L., Gu, J., *et al.* Construction of liver hepatocellular carcinoma-specific lncRNA–miRNA–mRNA network based on bioinformatics analysis. **16**(4), e0249881 (2021). 10.1371/journal.pone.0249881 PMC8051809 33861762

[42] Li J, Liu X, Cui Z, Han G (2020). Comprehensive analysis of candidate diagnostic and prognostic biomarkers associated with lung adenocarcinoma. Med. Sci. Monitor Int. Med. J. Exp. Clin. Res..

[43] Zhou Y, Wang Y, Cheng J, Zhang Y, Cai W (2021). Bioinformatics analysis revealed potential tumor suppressors (KLF4/CGN), oncogenes (SHH/LIF) and biomarkers of Asian stomach adenocarcinoma. Yangtze Med..

[44] Li Y, Li L (2020). Bioinformatic screening for candidate biomarkers and their prognostic values in endometrial cancer. BMC Genet..

[45] Chen P, Hsu W-H, Han J, Xia Y, DePinho RA (2021). Cancer stemness meets immunity: From mechanism to therapy. Cell Rep..

